# A Reappraisal of the Utility of L-012 to Measure Superoxide from Biologically Relevant Sources

**DOI:** 10.3390/antiox12091689

**Published:** 2023-08-30

**Authors:** Stephen Haigh, Zach L. Brown, Mitch A. Shivers, Hunter G. Sellers, Madison A. West, Scott A. Barman, David W. Stepp, Gabor Csanyi, David J. R. Fulton

**Affiliations:** 1 Vascular Biology Center, Medical College of Georgia at Augusta University, 1460 Laney Walker Blvd, CB 3316, Augusta, GA 30909, USA; 2 Department of Pharmacology and Toxicology, Medical College of Georgia at Augusta University, 1460 Laney Walker Blvd, CB 3316, Augusta, GA 30909, USA; 3 David Fulton Vascular Biology Center, Department of Pharmacology and Toxicology, Medical College of Georgia at Augusta University, 1460 Laney Walker Blvd, CB 3316, Augusta, GA 30909, USA

**Keywords:** superoxide, ROS, L-012, NADPH oxidase

## Abstract

The detection of superoxide anion (O_2_^●−^) in biological tissues remains challenging. Barriers to convenient and reproducible measurements include expensive equipment, custom probes, and the need for high sensitivity and specificity. The luminol derivative, L-012, has been used to measure O_2_^●−^ since 1993 with mixed results and concerns over specificity. The goal of this study was to better define the conditions for use and their specificity. We found that L-012 coupled with depolymerized orthovanadate, a relatively impermeable tyrosine phosphatase inhibitor, yielded a highly sensitive approach to detect extracellular O_2_^●−^. In O_2_^●−^ producing HEK-NOX5 cells, orthovanadate increased L-012 luminescence 100-fold. The combination of L-012 and orthovanadate was highly sensitive, stable, scalable, completely reversed by superoxide dismutase, and selective for O_2_^●−^ generating NOXes versus NOX4, which produces H_2_O_2_. Moreover, there was no signal from cells transfected with NOS3 (NO^●^) and NOS2(ONOO^−^). To exclude the effects of altered tyrosine phosphorylation, O_2_^●−^ was detected using non-enzymatic synthesis with phenazine methosulfate and via novel coupling of L-012 with niobium oxalate, which was less active in inducing tyrosine phosphorylation. Overall, our data shows that L-012 coupled with orthovanadate or other periodic group 5 salts yields a reliable, sensitive, and specific approach to measuring extracellular O_2_^●−^ in biological systems.

## 1. Introduction

The luminol derivative, L-012, was first reported in 1993 as a sensitive approach for the detection of superoxide anion (O_2_^●−^) from cells, and in the years since, there have been numerous papers utilizing this approach to detect O_2_^●−^ [1]. While many of these studies successfully used L-012 to measure O_2_^●−^, others have raised concerns over the perceived lack of specificity [2,3,4,5]. Upon closer inspection of the methods employed, it is clear that not only a standardized approach for measurement of L-012 chemiluminescence is lacking, but in several studies a major problem lies with the different cofactors used to magnify the L-012 signal.

One of the co-factors combined with L-012 is horseradish peroxidase (HRP), which utilizes hydrogen peroxide (H_2_O_2_) and other reactive oxygen species (ROS) for activation [2]. While the use of HRP is a standard approach for the detection of H_2_O_2_ using luminol and other substrates [6,7], its combination with L-012 has not been used as extensively. Nor is it clear that this combination is optimal, as luminol plus HRP is not considered to be selective for O_2_^●−^ but can also react with H_2_O_2_, peroxynitrite (ONOO^−^), hydroxyl radical (OH^●^) and nitric oxide (NO^●^) [6,8,9]. The use of orthovanadate as a cofactor for L-012 was first reported by Sohn, et al. [10] as a sensitive method to detect O_2_^●−^ production in aortic rings. They showed that the combination of orthovanadate with L-012 increased signal sensitivity by a factor of 17 over L-012 alone. However, they found that orthovanadate could also have off-target effects on tyrosine phosphorylation [11], which we will address in this study.

Reactive oxygen species are important molecules in biological systems [12]. ROS are produced in the mitochondria, where electron transport chain uncoupling causes O_2_^●−^ generation [13], as well as from families of dedicated enzymes, such as the NADPH oxidases (NOXes), that specifically create ROS in a time and location-dependent manner [14]. There are five distinct NOX isoforms, as well as two related members (DUOXS). All are transmembrane enzymes that span the membrane six times and convert oxygen to O_2_^●−^ in a heme-dependent manner via reduction of NADPH. Different NOX isoforms have specificity towards the emission of O_2_^●−^ (NOX1,2,3 and 5) versus H_2_O_2_ or generation (Nox4 and DUOXes) [15,16]. NOX4 and the DUOXes initially synthesize O_2_^●−^ however, due to the structure of respective active sites, O_2_^●−^ is rapidly converted to H_2_O_2_ [17]. NOX enzymes were initially discovered in phagocytotic cells where the oxidative burst is used to kill pathogens [18]. However, since these initial discoveries, NOX enzymes have been found in various tissues and in particular within the vasculature in cardiovascular disease conditions [19]. The Nitric Oxide Synthase (NOS) family of enzymes includes nNOS (neuronal NOS), iNOS (inducible NOS), and eNOS (endothelial NOS), and they share a similar catalytic model using NADPH to initially generate O_2_^●−^ but synthesize another radical species, NO, from substrate L-arginine [18]. The NOS enzymes, iNOS in particular and eNOS when BH4 levels are low (uncoupled eNOS), can emit a combination of NO and O_2_^●−^ leading to ONOO^-^ production [20].

The goal of this study was to optimize reaction conditions for the high sensitivity detection of O_2_^●−^ using L-012 and evaluate its specificity against enzymes specifically producing O_2_^●−^, H_2_O_2_, NO^●,^ and ONOO^−^ in the physiological range from intact cells.

## 2. Methods

### 2.1. Cell Culture and DNA Constructs

HEK293 cells were grown at 37° in 5% CO_2_ as described [21]. Cells were transfected using Lipofectamine 2000 according to the manufacturer’s protocol (ThermoFisher, Waltham, MA, USA). Briefly, 1000 ng of DNA was combined with lipofectamine 2000 (at a ratio of 1:2) in 200 uL of Opti-MEM (ThermoFisher) for 15 min. DNA complexes were then added to a 12-well plate containing HEK293A cells at 80% confluency in an Opti-MEM medium. At 3 h post-transfection, the medium was changed to DMEM (Invitrogen) with 10% FCS. The NOX5 HEK293 cell line was used as described in [15]. Plasmid DNA encoding RFP, eNOS, iNOS, NOX1, NOXA1, NOXO1, NOX4-v5, and HA-NOX5 was previously described in [15,21,22,23,24,25,26].

### 2.2. ROS Measurements

L-012 (Wako Chemicals, VA, USA) (Table S.1) was dissolved in DMSO at 200 mM and briefly sonicated to aid complete dissolution. Depolymerized sodium orthovanadate was made as previously described [27]. Phenazine methosulfate (5-Methylphenazinium methyl sulfate, CAS 299-11-6) was obtained from Sigma and combined with NADH to generate superoxide anion as shown previously [15]. Ammonium Nb-oxalate hydrate (Sigma-Aldrich, St. Louis, MO, USA) was solubilized at 200 mM in H_2_O and used at 1 mM unless otherwise indicated. HEK293A cells were used due to their ease of transfection and low ROS and RNS background due to the lack of endogenous ROS or RNS-producing enzymes. In brief, HEK293 cells were transfected on day 1, subcultured into a white 96-well plate (Corning^TM^, Corning, NY, USA) on day 2, and chemiluminescence measured on day 3 using a BMG Omega Star plate reader. Fifteen minutes prior to measurements, cell media was changed to serum-free, phenol-free, low-glucose DMEM (Sigma-Aldrich) containing 400 µM L-012 and 1 mM activated sodium orthovanadate, unless otherwise indicated. (Supplementary Method S.2) Hydrogen peroxide was measured using the Amplex™ Red Hydrogen Peroxide/Peroxidase Assay Kit (ThermoFisher) per manufacturer protocols. 2,4-diamino-6-hydroxypyrimidine (DHAP, Sigma-Aldrich) was added to cells at a concentration of 10 mM to deplete BH4 and uncouple NOS 24 h prior to measurements by chemiluminescence [28]. Catalase (CAT, 800 u/mL) and superoxide dismutase (SOD, 100 u/mL) were obtained from Sigma-Aldrich, dissolved in PBS, and used to specifically metabolize H_2_O_2_ and O_2_^●−^, respectively.

### 2.3. RNS Measurements

Furthermore, 48 h after transfection, media was collected, ethanol was added at 2 parts to 1 part of the media, and samples were chilled at −80 °C for at least 30 min. Samples were then centrifuged (20,000× *g*) and the supernatant assayed for NO_2_^−^ (the stable breakdown product of NO^●^ in aqueous media) using ozone chemiluminescence (Sievers 280i Nitric Oxide Analyzer, Sievers, CO, USA) as previously described [26]. Peroxynitrite was measured in media using dihydrorodamine123 (DHR123) as previously described [24,29].

### 2.4. Western Blot Analysis

Cells were washed with phosphate-buffered saline and lysed with 50 mM Tris-HCl, pH 6.8, 2% SDS, 30% glycerol, 6% β-mercaptoethanol, and 0.02% bromphenol blue for 3 min. Samples were then collected, sonicated briefly, and boiled at 95 °C for 5 min, followed by centrifugation at 16,000 g for 5 min. Samples were size-fractionated using either 7% or 10% SDS-polyacrylamide gels, transferred to nitrocellulose membranes (0.45 um pore size), and immunoblotted with relevant antibodies (Table S.3).

### 2.5. Statistics

Significance was calculated in GraphPad Prism 9 using Analysis of Variance followed by a post-hoc Tukey Kramer analysis or a *t*-test as indicated. Some figures were transformed with a natural log prior to statistics being run due to incomparable variance.

## 3. Results

**Figure 1:**
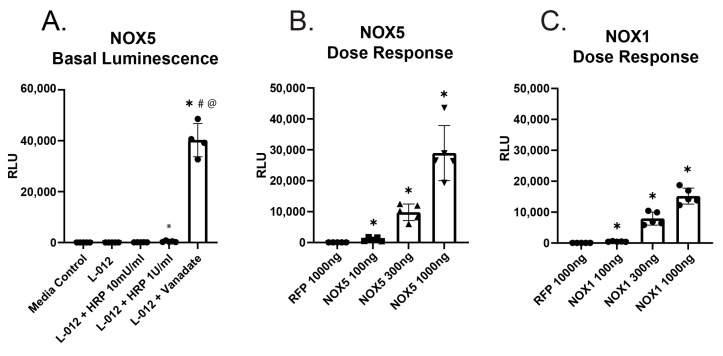
Detection of superoxide anion from enzymatic sources using L-012. (**A**). HEK293A. Cells stably transfected with Nox5 were split into a 96-well plate. 24 h later, superoxide anion was measured using L-012 (400 µM) in cells treated with either orthovanadate (1 mM) or HRP (10 mU/mL or 1 U/mL). (* indicates *p* < 0.05 compared to the Media group, # indicates *p* < 0.05 compared to the L-012 group, and @ indicates *p* < 0.05 compared to L-012+1uHRP group per ANOVA post hoc Tukey Kramer analysis) (**B**,**C**). Nox1 or Nox5, respectively, were transfected in a dose-dependent manner (100, 300, or 1000 ng of plasmid DNA) into cells. Chemiluminescence was measured as described previously. (* indicates *p* < 0.05 compared to control per log adjusted ANOVA post-hoc Tukey Kramer analysis).

We first compared the efficacy of orthovanadate as a cofactor for L-012 versus HRP in measuring ROS production from HEK-NOX5 cells. As shown in Figure 1A, the combination of L-012 and orthovanadate resulted in a ~3000-fold increase in signal compared to L-012 alone, while 10 mU/mL HRP gave only a slight increase in signal compared to L-012 (Figure 1A). To assess the ability of the L-012/orthovanadate combination to detect O_2_^●−^ production in a dose-dependent manner, HEK 293A cells were transfected with either NOX5 or NOX1 (along with co-factors NOXO1 and NOXA1) using increasing amounts of plasmids, 100 ng, 300 ng, or 1000 ng per well, in a 12-well plate. There was a clear relationship between the amount of plasmid transfected and the increase in signal observed (Figure 1B,C).

**Figure 2:**
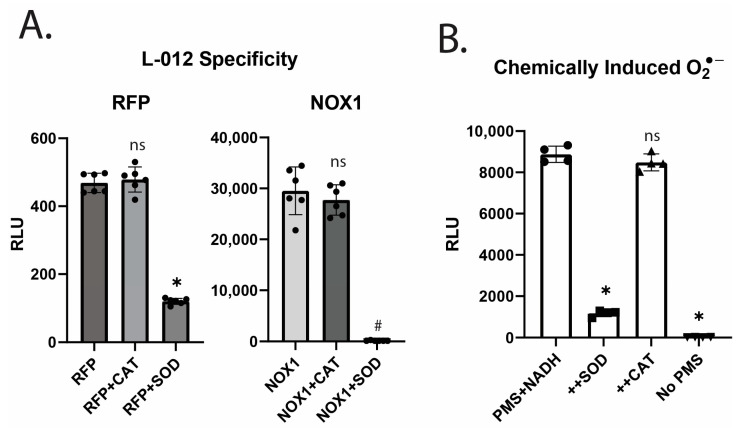
Detection of superoxide anion in a dose- and SOD-dependent manner using L-012. (**A**). HEK293A cells were transfected with RFP or Nox1 (A1, O1 subunits as well). The superoxide anion was measured as described previously. SOD (100 u/mL) or catalase (100 u/mL) was added to the wells 5 min prior to measurement. (* indicates *p* < 0.05 to RFP control, # indicates *p* < 0.05 to NOX1 control per ANOVA post hoc Tukey Kramuer analysis) (**B**). 96 well plates were loaded with PMS (15 µM) and treated with SOD (100 u/mL), CAT (800 u/mL), or control (no PMS), followed by NADH (73 µM final) injection, and luminescence was measured using L-012 (400 µM) and orthovanadate (1 mM). Measurements were performed every 30 s for 15 min. (* indicates *p* < 0.05 compared to control per ANOVA post hoc Tukey Kramer analysis, ns indicates not significant).

We next assessed the specificity of the combination of L-012 and orthovanadate in detecting O_2_^●−^. HEK293 cells transfected with NOX1 (and NOXO1, NOXA1) or a control RFP plasmid were treated with either the O_2_^●−^ scavenger superoxide dismutase 1 (SOD) or the scavenger of H_2_O_2_, catalase (CAT), and the resulting chemiluminescence signal was measured. The NOX1-dependent increase in chemiluminescence was highly sensitive to inhibition by SOD; however, CAT had no effect (Figure 2A). To assess L-012 specificity in a manner independent of NOX enzymes, O_2_^●−^ was generated via the interaction of phenazine methosulphate (5-methylphenazinium methyl sulfate, PMS) with NADH [15]. The chemiluminescence resulting from this reaction showed similar specificity to enzymatically generated O_2_^●−^, with signals sensitive to SOD but not altered by CAT (Figure 2B). These data suggest that L-012 with orthovanadate is preferentially sensitive to O_2_^●−^.

**Figure 3:**
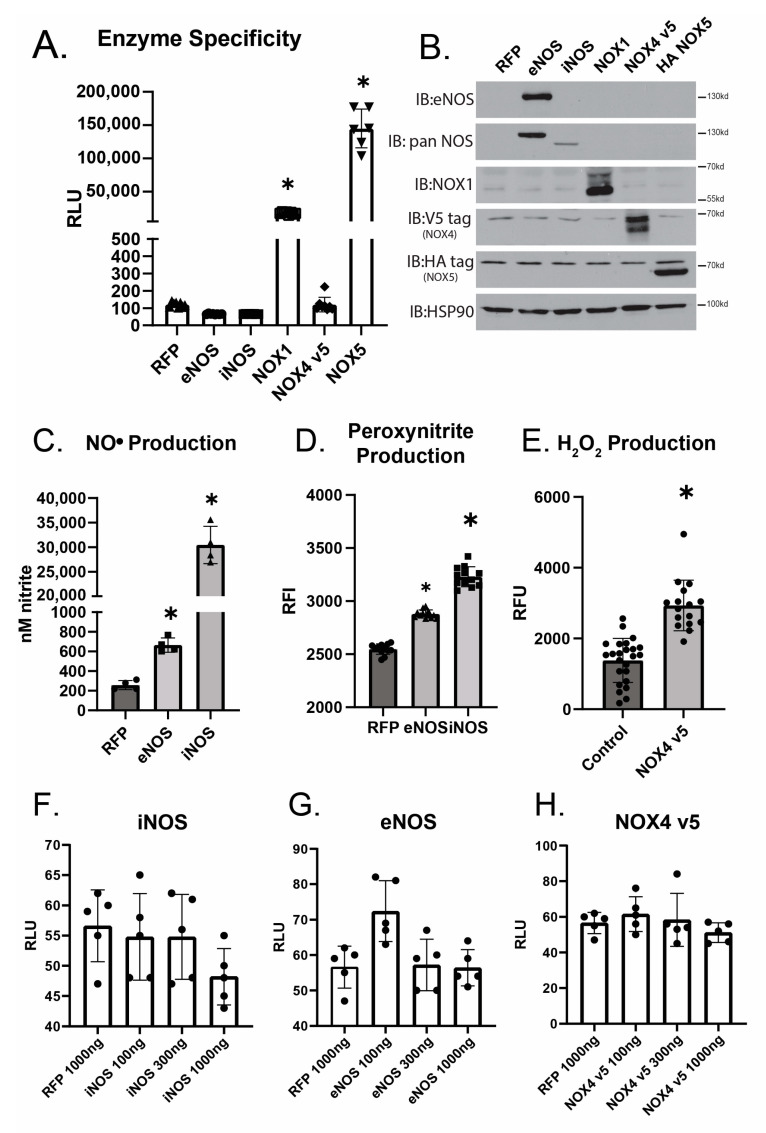
L-012 does not detect enzymatic sources of RNS or H_2_O_2_. (**A**). HEK293A cells were transfected with RFP, eNOS, iNOS, Nox1, Nox4-v5, or NOX 5 and split (24 h) into a 96-well plate. 48 h after transfection, the medium was changed to serum-free, phenol-free, low-glucose DMEM with 400 µM L-012 and 1 mM orthovanadate. The measurement was recorded 15 min after the media change. (**B**). Cells from (**B**) were also split into a 24-well plate. At 48 h post-transfection, cells were lysed, run for WB, and immunoblotted for the indicated targets. (* indicates *p* < 0.05 compared to the RFP group). (**C**). Cells were transfected with eNOS or iNOS (1000 ng/well), and media was collected after 48 h and measured for NO_2_^−^. (**D**). Cells transfected with eNOS or iNOS were exposed to DHR (5 um) for 60 min. Data expressed as compared to control, untransfected cells. (**E**). Cells were transfected with NOX4-V5 and assayed 48 h later for H_2_O_2_ production. (* indicates *p* < 0.05 and *p* < 0.0001, respectively, per ANOVA post hoc Tukey Kramer analysis). (**F**–**H**). iNOS, eNOS, and Nox4-v5 (tag) were transfected in a dose-dependent manner (100, 300, and 1000 ng of plasmid DNA), and chemiluminescence was measured as described previously.

To further investigate whether L-012 plus orthovanadate is specific for O_2_^●−^, the effects of biologically relevant enzymatic sources of reactive nitrogen species (RNS) and H_2_O_2_ were next examined. HEK293 cells were transfected with expression plasmids encoding a range of enzymes that produce different ROS and RNS: (1) O_2_^●−^ (NOX1, NOX5), (2) H_2_O_2_ (NOX4), and (3) NO/ONOO^−^ (eNOS, iNOS), versus a control plasmid expressing RFP (Figure 3A). Using the combination of L-012 and orthovanadate, only the membrane spanning NOX1 and NOX5, which produce O_2_^●−^, showed significantly increased chemiluminescence despite robust protein expression of all of the transfected enzymes (Figure 3B). While there was no increase in L-012 chemiluminescence, there were significant increases in detectable NO^●^ (Figure 3C), peroxynitrite (Figure 3D), and H_2_O_2_ (Figure 3E) that accompanied the expression of relevant enzymes. To investigate the possibility that higher amounts of RNS or H_2_O_2_-generating enzymes could overcome a potentially lower sensitivity threshold, we increased the amounts of iNOS, eNOS, or NOX4 transfected. Increased expression of iNOS, eNOS, or NOX4 resulted in no significant changes in the L-012 plus orthovanadate signal as compared to baseline (Figure 3F,G).

**Figure 4:**
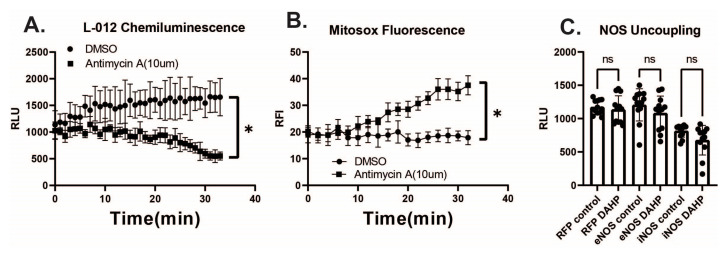
L-012 does not detect intracellular ROS. (**A**). HEK 293A cells treated with control (DMSO) or Antimycin A (10 µM) using L-012 luminescence as previously described. (**B**). HEK293A cells were treated with Antimycin A (10 µM) or DMSO control, and fluorescence intensity was detected using the Mitosox Red assay kit. (**C**). Cells were transfected as indicated, split into a 96 well plate, and treated with DAHP (10 mM) for 24 h prior to measurement to deplete BH4. (* indicates *p* < 0.05 per *t*-test (at 30 min) analysis, ns indicates not significant).

Given the relative cellular impermeability of orthovanadate [30] and the ability of extracellular SOD to quench almost the entire signal from L-012 and orthovanadate, our assumption is that we are measuring extracellular O_2_^●−^. To confirm this, we generated intracellular ROS using antimycin A to induce electron transport chain (ETC) dysfunction in the mitochondria and generate ROS including O_2_^●−^ [31]. In cells treated with antimycin A, ROS were undetectable using L-012 and orthovanadate in this experiment and instead showed a decrease in chemiluminescence over time compared to the vehicle (DMSO) control (Figure 4A). To ensure that the mitochondria were indeed uncoupled and generating increased ROS, MitoSOX red was utilized to detect ROS in the mitochondria at the same dose and timepoints following antimycin A exposure (Figure 4B). Given the ability of NOS enzymes such as eNOS in the absence of tetrahydrobiopterin (BH4) to generate intracellular O_2_^●−^ instead of NO^●^ [20] we next treated cells expressing eNOS and iNOS with DAHP, which inhibits GTP cyclohydrolase, the rate-limiting step in BH4 production. BH4 was depleted for 24 h using DAHP, but there was no increase in chemiluminescence from L-012 plus orthovanadate (Figure 4C). These data suggest that L-012 and orthovanadate are limited to the measurement of extracellular O_2_^●−^.

**Figure 5:**
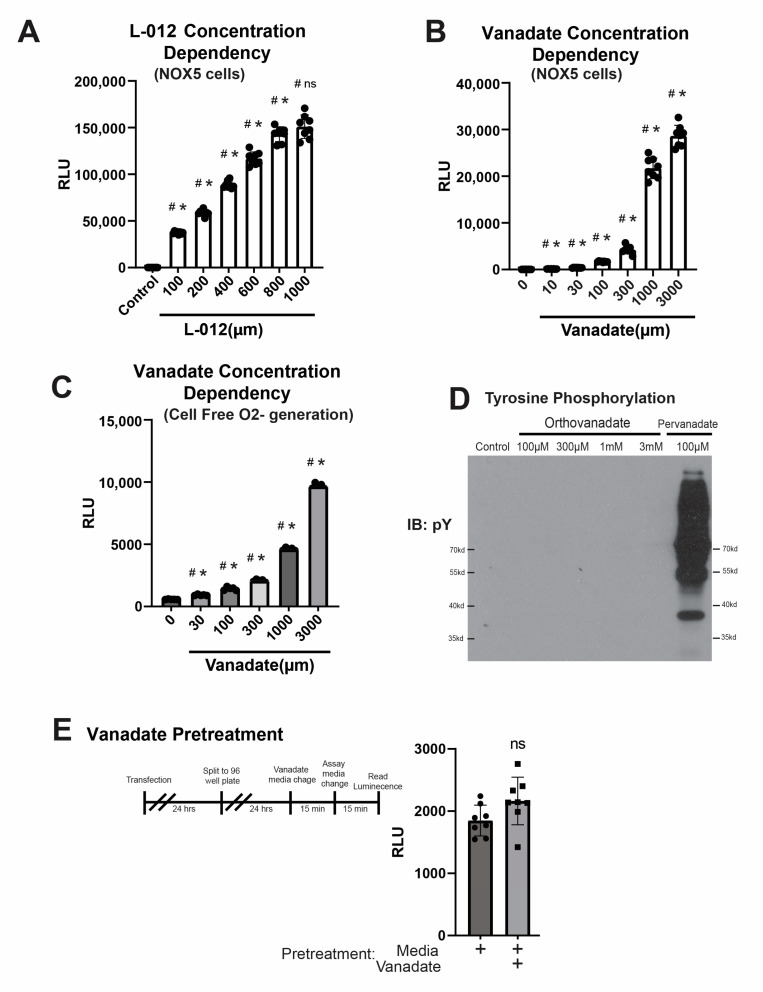
Orthovanadate increases the L-012 signal in a tyrosine phosphatase-independent manner. (**A**). HEK293A cells were transfected with Nox5 and split as previously described. Cellular luminescence was then measured with 1 mM orthovanadate and the above-indicated concentration (uM) of L012. (**B**). HEK293A cells were transfected with Nox5 and split as previously described. Cellular luminescence was then measured with L012 (400 µM) and the above-indicated concentration (mM) of orthovanadate. (**C**). Wells loaded with PMS were injected with NADH and measured using L-012 (400 µM) and the above-indicated dose of orthovanadate. (**D**). Cells were treated with the indicated concentrations of orthovanadate for 15 min before being lysed. Protein lysates were run on a WB and immunoblotted using a pan-phosphotryosine antibody. (**E**). Cells transfected with Nox1 were assayed for chemiluminescence, and the media was changed 30 min prior to either orthovanadate-containing media or not. Further, there was a group where no media was changed. (* indicates *p* < 0.05 compared to the previous (lower concentration) group; # indicates *p* < 0.05 compared to the untreated control, ns indicates not significant)

**Figure 6:**
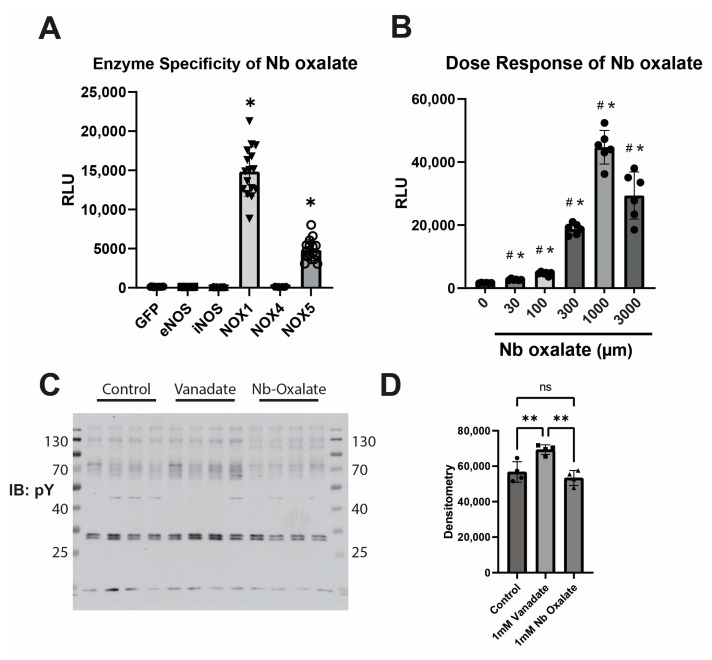
Nb-oxalate is a viable alternative for orthovanadate in combination with L-012. (**A**). HEK293A cells were transfected with the indicated construct, split, and measured as previously described using 1 mM ammonium Nb-oxalate and L-012 instead of orthovanadate with L-012. (**B**). HEK293A cells transfected with NOX5 were split as previously described and measured using the indicated concentration of Nb-oxalate. (**C**,**D**). HEK293A cells were treated with control media or media containing either 1 mM orthovanadate or 1 mM Nb-oxalate for 30 min prior to lysis. Protein lysates were run on a WB and immunoblotted using a pan-phosphotryosine antibody. (**C**) Densitometry was calculated using ImageJ software (**D**). (# indicates *p* < 0.05 compared to the previous (lower concentration) group, * indicates *p* < 0.05 compared to the untreated control, ns indicates not significant.)

To assess the degree of optimization of our assay conditions, HEK293 cells stably expressing NOX5 were treated with 1 mM orthovanadate while the L-012 concentration was varied and the peak signal was observed at 800 µM (Figure 5A). Similarly, in HEK-NOX5 cells, L-012 was held constant at 400 uM and the orthovanadate concentration varied. The concentration-response relationship with orthovanadate was quite distinct from that of L-012, being much steeper with a threshold of 1000 µM and a maximum at 3000 µM (Figure 5B). Orthovanadate is a potent tyrosine phosphatase inhibitor, and to exclude any potential post-translational effects on NOX enzyme function or other signaling events, we repeated the orthovanadate dose dependence using the cell-free O_2_^●−^ generator, PMS (Figure 5C). The ability of orthovanadate to increase the signal from PMS was equivalent to that observed in cells. While orthovanadate is largely cell-impermeable, pervanadate is considered to be more hydrophobic and cross cell membranes [32] to robustly increase intracellular tyrosine phosphorylation. To compare overall tyrosine phosphorylation, HEK 293A cells were treated for 15 min with varying doses of orthovanadate or a low dose of pervanadate. Orthovanadate had a minor effect on phosphorylated tyrosine levels, especially in perspective with the very strong increases evoked by pervanadate (Figure 5D and Figure 6C,D with higher exposure). To exclude an effect of orthovanadate on tyrosine phosphorylation that altered O_2_^●−^ generation, cells were pretreated for 30 min with orthovanadate prior to following our standard protocol as shown. No difference in signal between the treated and control groups was observed, suggesting the effects of orthovanadate are acute and mediated by interaction with L-012 (Figure 5E).

We next investigated other approaches to avoid the potential inhibition of tyrosine phosphatases by orthovanadate by considering the possibility that other periodic group 5 transition metal salts could have similar properties in enhancing L-012 chemiluminescence while remaining specific to O_2_^●−^. From these possibilities, we selected niobium and tantalum to explore. We encountered poor solubility with structurally similar compounds, including sodium niobate (NaNbO_3_) or sodium tantalate (NaO_3_Ta), that limited applicability. To get around the solubility issue, we used a different compound called ammonium niobium-oxalate (Nb-oxalate) (C_6_H_4_NNbO_12_), which has a very high solubility in H_2_O. We found that the performance of Nb-Oxalate was very similar to that of orthovanadate in both its specificity for O_2_^●−^ (Figure 6A) and its dose range (Figure 6B). Furthermore, we were able to show that there was no apparent inhibition of tyrosine phosphatases, as indicated by a lack of increased phosphotyrosine staining (Figure 6C,D).

## 4. Discussion

Herein, we provide a reappraisal of the use of L-012 in combination with orthovanadate as a method that can detect O_2_^●−^ from biologically relevant sources with very high sensitivity, scalability, and reasonable specificity. We recommend a standard protocol of 400 µM L-012 and 1 mM orthovanadate, which is the midpoint between the maximal sensitivity seen with 800 µM L-012 and 3 mM orthovanadate and more likely to provide a balance between the optimal ability to detect O_2_^●−^ and the potential for non-specific actions. The combination of L-012 and orthovanadate readily detected the activity of the O_2_^●−^ producing NOX1 and NOX5 from cells, but not the H_2_O_2_ emitting NOX4, the NO^●^ producing eNOS and iNOS, uncoupled eNOS, or mitochondrial uncoupling agents.

Our data combining L-012 with orthovanadate supports the specific and sensitive detection of O_2_^●−^ from cells; however, others have reported that L-012 is nonspecific and detects H_2_O_2_ [2]. While it has been demonstrated quite convincingly that H_2_O_2_ can be detected using L-012, HRP was used as a cofactor to increase signal intensity. In contrast to this data, no signal was detectable with the use of orthovanadate as a cofactor when used to detect biological sources of H_2_O_2_ (NOX4). Further, the addition of catalase had no effect on the L-012 signal, unlike previous reports using HRP [2], but the signal was readily reversed by the addition of superoxide dismutase. These data suggest that while L-012 is a luminol derivative [1,6], the use of HRP provides only a modest increase in sensitivity and a loss of specificity for superoxide anion. Other studies suggest that L-012 can react with nitric oxide and peroxynitrite as the main signal-inducing species [3,33]. Using biologically relevant enzymatic sources of these species (iNOS, eNOS), no increase in signal stemming from the generation of nitric oxide or peroxynitrite from cells was observed using L-012 and orthovanadate as a cofactor. The presence of nitric oxide and peroxynitrite was confirmed using the DHR assay, which can detect ONOO^−^ and NO^●^ specific chemiluminescence [29]. It is possible that other radial species contribute to L-012 chemiluminescence at low frequencies versus superoxide anion and that higher concentrations of reactive nitrogen or oxygen radicals, such as with isolated enzymes or chemicals, can increase this frequency.

Given the high reactivity, short half-life, and low abundance of superoxide anion, a major advantage of tools to measure superoxide anion from biological sources is their high sensitivity. We found that the combination of L-012 and orthovanadate boosted sensitivity by greater than 3000-fold over L-012 alone without compromising its selectivity. Compared to other approaches such as EPR or DHE staining, L-012 plus orthovanadate confers a significant advantage in measuring signal to noise [34]. Many studies have used L-012 in the absence of orthovanadate or other co-factors which may have compromised the ability to detect low levels of superoxide anion [35,36]. Exactly how orthovanadate amplifies L-012 chemiluminescence is not completely clear. Interestingly, L-012 and orthovanadate were able to measure superoxide anion in a scalable fashion in response to a stimulus or at baseline in proportion to increasing expression of a superoxide anion synthesizing enzyme and yield a signal that was sustained for over an hour [23,24]. It is not known what fraction of the pool of L-012 interacts with superoxide anion, but the ability to measure a sustained signal suggests it is a small percentage. How this changes with time and affects assay sensitivity over time are important considerations. Earlier studies looking at the O_2_^●−^/vanadate interaction reported that it can form a peroxyvandyl complex, which can oxidize electron donors [37].

Despite the numerous advantages of the combination of L-012 and orthovanadate in measuring superoxide anion, there are some limitations worthy of discussion. Orthovanadate is perhaps best known as an inhibitor of tyrosine phosphatases [27] and is routinely added to buffers for cell lysis. While effective in lysed cells, orthovanadate has limited cellular permeability, which reduces its ability to inhibit phosphatases in intact cells [38]. Compared to pervandate, which can cross cellular membranes and is more effective at inhibiting tyrosine phosphatases, we found that orthovanadate was comparatively weak, with almost undetectable increases in tyrosine phosphorylation over the short term and minor changes over longer periods of time. Others have shown greater increases in protein tyrosine phosphorylation [10] with extracellular orthovanadate, and this could be more significant with higher concentrations of orthovanadate, longer periods of time, or different cell types. To rule out the potential effects of orthovanadate on NOX activity versus its effects on L-012 chemiluminescence, we used PMS to generate superoxide anion in the absence of NOX enzymes and compared the activity of NOX expressing cells pretreated with orthovanadate or not. We found that orthovanadate was also very effective in enhancing the L-012-dependent chemiluminescence from PMS-generated superoxide anion and that pretreatment of cells expressing NOX enzymes with pervanadate did not modify the signal. These findings suggest that the effects of orthovanadate on cells are largely minor and do not play a significant role in the signal detected. To further exclude any effects on tyrosine phosphorylation, we also investigated the suitability of other Group 5 transition metal salts, niobium, and tantalum salts, in combination with L-012 to detect superoxide anion. We identified Nb-oxalate as a potential alternative due to its availability and high solubility. Nb-oxalate seems to work very similarly to orthovanadate but either is completely incapable of cell entrance or does not inhibit tyrosine phosphatase, as we saw no effect on overall tyrosine phosphorylation with this compound versus minor effects with orthovanadate. Based on our data, Nb-oxalate should be considered further for use in combination with L-012, especially by those investigating disease pathways or the post-translational regulation of NOX enzymes. Another limitation of using orthovanadate to measure superoxide anion is its inability to detect intercellular sources of O_2_^●−^. Mitochondria are an important source of intracellular superoxide anion, and uncoupling of the ETC resulted in elevated intracellular superoxide anion that was detectable with mitoSOX but not L-012. The extracellular measurement of superoxide anion results from the localization of the NADPH oxidase in the plasma membrane of the cell. NOX enzymes are also found on intracellular membranes [39,40] and the relative ability of intracellular versus extracellular NOXes to generate superoxide anion is not known. While this is a limitation of L-012, extracellular superoxide anion has a number of important roles, such as NO^●^ catabolism, LDL oxidation, and the immune cell oxidative burst.

Our study supports the use of 400 µM L-012 and 1 mM orthovanadate as a sensitive approach to measuring extracellular superoxide anion. Greater sensitivity can be acquired with the use of higher concentrations of both L-012 and orthovanadate, but this raises the potential for unintended effects on cellular and enzyme function. We further propose the combination of L-012 and Nb-oxalate as an alternative to orthovanadate that has a comparable ability to enhance L-012 chemiluminescence with a reduced potential for protein tyrosine phosphorylation. Overall, the advantages of this approach, including the cost, accessibility with a luminescent plate reader or luminometer, high sensitivity, selectivity for superoxide anion, scalability, and low toxicity, outweigh the disadvantages, such as the inability to measure intracellular superoxide anion and the potential for minor post-translational changes in tyrosine phosphorylation.

## Data Availability

Data sharing is not applicable to this article due to data type.

## Supplementary Materials

The following supporting information can be downloaded at: https://www.mdpi.com/article/10.3390/antiox12091689/s1. Table S.1: Chemicals and solutions; Supplementary Method S.2: Chemiluminescent standard assay; Table S.3: Antibody list.
