# Supplementary material for: A Reappraisal of the Utility of L-012 to Measure Superoxide from Biologically Relevant Sources
Source: Antioxidants (Basel). 2023 Aug 30;12(9):1689. doi: 10.3390/antiox12091689 (PMC10525458; doi:10.3390/antiox12091689)
Supplement: Supplementary file 1 [file antioxidants-12-01689-s001.zip › antioxidants-2589245-SI.pdf]

Supplementary materials:

Table S.1 Chemicals and Solutions

Compound	Company	Product Number
L-012	Fujifilm Wako	120-04891
Superoxide Dismutase	Sigma-Aldrich	S5395-30KU
Sodium Orthovanadate	Sigma-Aldrich	S6508-10G
Ammonium Niobate(V) oxalate	Sigma-Aldrich	525839-50G
Catalase	Sigma-Aldrich	C9322-1G
DMEM	Sigma-Aldrich	D2902-10x1L
HRP	ThermoFisher	31490

Stock solutions:

L-012

L-012 is solubilized in DMSO at 200mM vortexed vigorously and sonicated(~10 seconds).

Orthovanadate:

1. Prepare a 200 mM solution of sodium orthovanadate.

2. Adjust the pH to 10.0 using either 1 N NaOH or 1 N HCl. The starting pH of the sodium orthovanadate solution may vary with lots of the chemical. At pH 10.0 the solution will be yellow.

3. Boil the solution until it turns colorless (approximately 10 minutes).

4. Cool to room temperature.

5. Readjust the pH to 10.0 and repeat steps 3 and 4 until the solution remains colorless and the pH stabilizes at 10.0.

6. Store the activated sodium orthovanadate as aliquots at -20C.

SOD:

SOD is solubilized at 30,000 units per mL in $H_2O$and stored as aliquots at -20C

Catalase:

Catalase is solubilized at 30,000 units per mL in H_2O and stored as aliquots at -20C

Ammonium Niobate:

Ammonium Niobate(V) is solubilized at 200mM in H_2O and stored as aliquots at -20C

DMEM media:

Low Glucose phenol free DMEM from Sigma-Aldrich(D2902-10x1L) was made per manufactures instructions with bicarb added at 3.7g/L, media was then filter sterilized and stored at 4C.

METHODs:

Supplementary Method S.2 Chemiluminescence standard assay:

1. HEK293A Cells were transfected with the relevant plasmids(lipofectamine 2000 per manufactures protocol)
2. 24 hrs later split to a 96 well cell culture treated plate(BRAND 781965) at 25000 cells/well in 100μL media.
3. 24 hrs after splitting cell media is suctioned out and replaced with DMEM media containing 1mM Orthovanadate and 400μM L-012 at 100μL/well
4. 15 min post media change luminescence is read using a BMG Polarstar Omega.

Table S.3 Antibody list

Antibody	Company	Product Number
Purified Mouse Anti-eNOS/NOS Type III	BD Biosciences	610297
V5 Tag Monoclonal Antibody (SV5-Pk1)	ThermoFisher Scientific	R960-25
Phosphotyrosine Monoclonal Antibody (pY20)	ThermoFisher Scientific	#03-7700
HA-Tag (C29F4)	Cell Signaling	3724S
Anti- NOX1	Sigma-Aldrich	SAB4200097
iNos (pan)	Cell Signaling	2977S
anti-HSP90	BD Biosciences	610419
